# Establishment of the *Drawida gisti* species complex and description of a new species from northeastern China (Oligochaeta, Moniligastridae)

**DOI:** 10.3897/zookeys.1293.204408

**Published:** 2026-10-01

**Authors:** Saiyu Qiao, Jiangni Li, Yufeng Zhang, Nonillon M. Aspe, Huifeng Zhao

**Affiliations:** 1 Hebei Key Laboratory of Animal Diversity, Langfang Normal University, Langfang 065000, China Hebei Key Laboratory of Animal Diversity, Langfang Normal University Langfang China; 2 Hebei Key Laboratory of Zoological Systematics and Application, College of Life Sciences, Hebei University, Baoding, 071002, China Hebei Key Laboratory of Zoological Systematics and Application, College of Life Sciences, Hebei University Baoding China; 3 College of Environment and Life Sciences, Mindanao State University at Naawan, Naawan 9023, Misamis Oriental, Philippines College of Environment and Life Sciences, Mindanao State University at Naawan Naawan Philippines

**Keywords:** Annelida, mitogenome, new species, species group

## Abstract

The family Moniligastridae Claus, 1880 remains taxonomically poorly resolved, and its actual species diversity is likely severely underestimated. Despite its ecological significance as an East Asian endemic species, *Drawida gisti* Michaelsen, 1931 has been studied almost exclusively from non-taxonomic perspectives. Consequently, its species boundaries remain poorly understood. Here, we formally establish the *Drawida gisti* species complex (DiSC), comprising eight morphologically and geographically related species. We employed an integrative taxonomic approach combining morphology and molecular data to examine specimens of DiSC from northeastern China, leading to the description of a new species, *Drawida xuqini***sp. nov**. The new species is characterized by a colorless body in alcohol, 3–5 gizzards, M-shaped prostates, a very short and uncoiled vas deferens, and thick penes with a swollen base and blunt, rounded apex. Phylogenetic analyses based on 13 protein-coding genes of the mitochondrial genome of *Drawida* employing two molecular species delimitation methods—Assemble Species by Automatic Partitioning (ASAP), and Bayesian Phylogenetics and Phylogeography (BPP)—recovered *D. xuqini***sp. nov**. as a monophyletic lineage, and COI genetic distances (19.4–22.0% between *D. xuqini***sp. nov**. and *D. gisti gisti*) confirmed the species status of *D. xuqini***sp. nov**. Additionally, we provide an updated redescription of *D. gisti gisti* based on newly examined specimens from multiple localities. Our findings highlight considerable external morphological plasticity in *D. gisti gisti*, which may likely be environmentally induced, and underscore the necessity of integrative approaches for accurate species delimitation in this group.

## Introduction

The family Moniligastridae Claus, 1880 comprises five genera and 193 species. Among them, *Drawida* Michaelsen, 1900 is the largest genus with 163 species and subspecies (Tiwari et al. 2025, 2026; Ardra et al. 2026; Fahis et al. 2026; Qiao et al. 2026a, 2026b), distributed across South, Southeast, and East Asia (Gates 1936, 1972; Easton 1981; Blakemore 2012; Blakemore et al. 2014; Anderson et al. 2017; Zhang et al. 2020; Narayanan et al. 2025a, 2025b; Zhang et al. 2026). Integrative taxonomic methods have become a standard for species delimitation due to widespread cryptic speciation of earthworms (Chang and James 2011; Han et al. 2024, 2026; Qin et al. 2025; Liu et al. 2026; Xu et al. 2026). Moniligastridae remains taxonomically poorly resolved with likely underreported cryptic diversity (Liu et al. 2025a, 2025b). Within this family, *Drawida gisti* Michaelsen, 1931, an ecologically significant East Asian endemic, has been studied almost exclusively from non-taxonomic perspectives (Wang et al. 2020; Yin et al. 2022; Liao et al. 2024), leaving its taxonomy strikingly understudied and its true species diversity potentially obscured.

*Drawida gisti* is distributed in eastern China (Gates 1939; Kobayashi 1940; Blakemore et al. 2014; Bi et al. 2025). Original description of the species is from Jinan (in Shandong Province) and Beijing (Michaelsen 1931), with type specimens deposited in the Hamburg Museum. It measures 77–151 mm length, 6 mm diameter with 180–190 number of segments and is characterized by a slender vas deferens emerging from the testis sacs, forming a tightly coiled loop within segment IX, entering segment X to produce a similar convolution, and finally leading to the proximal portion of the prostate gland. Chen (1933) described one subspecies, *D. gisti nanchangiana*, based on minor morphological differences in the penial chamber and prostate distinguished it from *D. gisti gisti*. *Drawida gisti anchingiana* was concurrently described but later elevated to species level as *D. anchingiana* by Kobayashi (1936). Later redescriptions of *D. gisti* by Gates (1935, 1939), Kobayashi (1938), and Chen (1959) were added more details on spermathecae, prostate, and the gizzard, yet these accounts remain incomplete by modern standards and lack molecular validation.

We examined other morphologically similar species recorded from the distributional range of *D. gisti gisti*. These taxa were only reviewed based on published literature, as type specimens are presumed lost: *Drawida cheni* Gates, 1935 (body length 122–177 mm, diameter 4 mm) is distributed in Jiangsu; *D. linhaiensis* Chen, 1933 (37–58 mm, 3–4 mm) in Zhejiang, *D. sinica* Chen, 1933 (50–95 mm, 2.2–3.2 mm) in Jiangxi (Chen 1933; Gates 1935), *D. sulcata* Zhong, 1986 (60–86 mm, 9.0–12.5 mm) in Yunnan, and *D. syringa* Chen, 1933 (50–100 mm, 2.5–3.0 mm) in Zhejiang. These species all occur in eastern and southwestern China, with their type localities situated in relatively geographic proximity, suggesting potential biogeographic affinity. Also, these species highly share similarities in external and internal morphological characters with *D. gisti gisti*, including the presence of a penis and penial chamber. We therefore hypothesize that they represent closely allied species of *D. gisti gisti*, and we formally establish the *Drawida gisti* species complex (DiSC), comprising *D. gisti gisti*, *D. anqingiana*, *D. gisti nanchangiana*, with *D. cheni*, *D. linhaiensis*, *D. syringa*, *D. sinica*, and *D. sulcata* (Table 1).

**Table 1.** Key morphological characteristics of the *Drawida gisti* species complex (NA: not available).

**Characters**	***D. xuqini* sp. nov**.	***D. gisti gisti***	***D. anchingiana***	***D. gisti nanchangiana***	***D. cheni***	***D. linhaiensis***	***D. sinica***	***D. sulcata***	***D. syringa***
**Color**	light gray	Red-brown	light blue-green	light gray, without pigment	light blue	Light blue-gray	light green	brown	dusky flesh
**Length**	48–87	48–66	56–80	55–98	122–177	37–58	50–95	60–86	50–100
**Width**	4.5–7.0	5.0–7.0	3.0–5.0	2.2–3.0	4.0	3.0–4.0	2.2–3.2	9.0–12.5	2.5–3.0
**Genital markings**	VII–XI, unpaired, irregular	absent or present VIII–XII	VII–XI, unpaired	VII–XIII, XIV, unpaired, numerous	7–10, irregular	8–10, irregular	8–11	absent	absent or present in VII–IX
**Clitellum**	X–XIII; Pale pink	IX/X–XIII/XIV	X–XIII	X–XIII	½IX–XIV,½XV	X–XIII, pale	X–XIII, pale white	NA	X–XII
**Penis**	10/11, large, rounded at tip, large at base	10/11, long, large at base gradually tapering to sharp	10/11, small	10/11, small, pointed at tip, large at base	10/11, broad triangular at base	10/11, large, transversely ovoid, rounded at tip	10/11, small, rounded or transversely flat at tip, large at base 0.18 mm	Conical	10/11 thick at base tapering at tip
**Form of male pores**	small pore on penis	longitudinal slit on penis	small pore on penis	paired	at tip of penis	10/11	small, at tip or slightly lateral of penis	10/11, oval or eye-shaped	NA
**Female pore**	11/12	11/12	11/12, transverse slit-like	paired	12; in ab line	11/12; in b line	11/12; in b line	11/12, Small crack-shaped	NA
**Spermathecae ducts**	medium, coiled	medium, coiled	medium, less coiled	coiled	long, coiled	slender, coiled	long, coiled	long, coiled	coiled
**Spermathecae Ampulla**	VIII	VIII	VII	VIII	VIII	VIII	VIII	VIII	VIII
**Spermathecae Atrium**	large, elliptical in 7/8	large, comma-shaped in 8	short, cylindrical in 7/8	thumb-like and small in 8	NA	in 7/8	zigzag, long tube in 7/8	cylindrical and slightly flattened	thick and short, stick-shaped
**Ovaries**	ovary chambers present, ovaries in XI–XII	ovary chambers present, ovaries in X	ovary chambers present, ovaries in XI	ovary chambers present, ovaries in XI	NA	ovary chambers present, ovaries in XI	ovary chambers present, ovaries in XI	ovary chambers present, ovaries in XI	NA
**Ovisacs**	XIII–XXI	X–XIII/XVI/XVIII	NA	XII–XIIII	XII, XIV, or XV	XVI–XX	X/XI/XII–XVI or XXII	NA	Extend to XXV
**Gizzard segments and position**	3–5; IX–XVI, XII/XIII–XVIII or XI–XVII	3; IX/X–XV/XVII/XVIII	2 or 3; XI–XIII	3; XII–XV	3; XIII–XV	3; XIII–XVI	3 or 4; XI–XIII	3, XI/XII–XIV	2–5
**Vas deferens**	short, uncoiled	extremely long, two coils	long, coiled	long, coiled	short, twisted	multi-coiled	extremely long, two coils	short coiled	NA
**Prostate**	M-shaped, white, 4.705–11.614 mm	Shape	slender, cylindrical, maximum 2 mm	Slender, short, club-shaped, 2.0–2.5 mm	multi-curved, 10–12 mm	large, long	long	smooth, long, 5.0–6.8 mm	short and broad, with thick glands on exterior

Here, we provide an updated redescription of *D. gisti gisti* based on the newly examined specimens. Moreover, we employ an integrative taxonomic approach to examine and analyze earthworm specimens belonging to the DiSC collected from northeastern China, leading to the delimitation of a new species, *Drawida xuqini* sp. nov. Mitochondrial genomes and three nuclear markers were sequenced and analyzed for *D. gisti gisti* and *D. xuqini* sp. nov.

## Material and methods

### Sampling

Specimens of the new species were collected from Weiyuan Park, Shanhaiguan District, Qinhuangdao Prefecture, Hebei Province, China. Specimens of *D. gisti gisti* were collected from Mentougou district in Beijing, Rizhao and Heze prefectures in Shandong Province, and Chengde Prefecture in Hebei Province, China (Table 2). All specimens were collected using hand-sorting and digging methods. Collected earthworms were immediately fixed and stored in 95% ethanol in the field. In the laboratory, all specimens were preserved at −20 °C in the Hebei Key Laboratory of Animal Diversity, Langfang Normal University, Hebei, China (**C-HLU**).

**Table 2.** List of *Drawida* species, incorporated into analysis (hyphen indicate not available).

**Specimen ID**	**Species**	**Location**	**Accession Number**				
			**COI**	**28S**	**AKAP17**	**FLAD1**	**Mitogenome**
DLS_35	*D. gisti gisti*	China: Beijing: Dongling Mountain	PX369606	PZ484227	PZ495527	PZ495535	PX369606
L668_09_01	*D. gisti gisti*	China: Hebei: Wuling Mountain	PZ462180	PZ484228	PZ495528	PZ495536	PZ462180
SDRZ_03	*D. gisti gisti*	China: Shandong: Rizhao	PZ462182	PZ484230	PZ495530	PZ495537	PZ462182
SDHZ04_01	*D. gisti gisti*	China: Shandong: Heze	PZ462181	PZ484229	PZ495529	PZ495534	PZ462181
BJMTG01_II_3	*D. gisti gisti*	China: Beijing: Mentougou	PZ462178	–	–	–	PZ462178
345R_01	*D. xuqini* sp. nov.	China: Hebei: Qinhuangdao	–	PZ484221	–	–	PZ168347
345R_33	*D. xuqini* sp. nov.	China: Hebei: Qinhuangdao	PZ486062	PZ484222	–	–	–
345R_34	*D. xuqini* sp. nov.	China: Hebei: Qinhuangdao	PZ486063	PZ484223	–	–	–
345R_38	*D. xuqini* sp. nov.	China: Hebei: Qinhuangdao	–	PZ484225	PZ495524	PZ495533	PZ462175
345R_39	*D. xuqini* sp. nov.	China: Hebei: Qinhuangdao	PZ486064	–	–	–	–
345R_40	*D. xuqini* sp. nov.	China: Hebei: Qinhuangdao	–	PZ484224	PZ495525	PZ495532	PZ462176
345R_42	*D. xuqini* sp. nov.	China: Hebei: Qinhuangdao	–	PZ484226	PZ495526	PZ495531	PZ462177
HNLN_GR_I1_02	*D. henanensis*	China: Henan: Luonling	PQ288546	–	–	–	PQ278996
HNLN_GR_I2_23	*D. henanensis*	China: Henan: Luonling	PQ288549	–	–	–	PQ278997
HNLN_GR_I2_34	*D. sinensis*	China: Henan: Luonling	PQ288559	–	–	–	PQ278998
HNLN_GR_I2_40	*D. sinensis*	China: Henan: Luonling	PQ288561	–	–	–	PQ278999
HNLN_GR_I3_01	*D. japonica* s. s.	China: Henan: Luonling	PQ288577	–	–	–	PQ279000
360R02	*D. japonica* s. s.	–	PZ168348	–	–	–	PZ168348
CQWX11_45	*D. wuxiensis*	China: Chongqing: Wuxi	PX552887	–	–	–	PX369605
CQWX05_37	*D. wuxiensis*	China: Chongqing: Wuxi	PX552881	–	–	–	PZ210301
CNWN02_01	*D. xinjiangensis*	China: Xinjiang: Shuanghe	PX978833	–	–	–	PZ099866
nxGXJ1_1_1	*D. xinjiangensis*	China: Ningxia: Xiji	PX978843	–	–	–	PZ210303
MN539609	*D. japonica* s. l.	China: Jiangsu: Nanjing	–	–	–	–	MN539609
–	*E. irregularis*	–	–	–	–	–	PZ168348
–	*M. anisodiverticulus*	–	–	–	–	–	PV591013

### Morphological analysis

External and internal characters were examined under a ZEISS stereomicroscope (ZEN v. 3.3 Pro). Measurements (to 0.1 mm) and dissections followed Blakemore et al. (2014) and Michaelsen (1892). Terminology follows Xu and Xiao (2011).

### Molecular data generation

Genomic DNA was extracted from posterior tissues (TIANGEN kit). Sanger sequencing targeted four markers: mitochondrial COI, nuclear 28S ribosomal RNA gene, 28S; A-kinase anchor protein 17A, AKAP17; flavin adenine dinucleotide synthetase 1, FLAD1 primers in Table 3. Sequences were assembled in MEGA 5 (Tamura et al. 2011); COI distances were calculated under Kimura two-parameter K2P model (Kimura 1980). For mitogenome reconstruction, high-throughput sequencing (150 bp paired-end, BGI Genomics) was performed on selected specimens, assembled with MitoZ v. 2.4 (Meng et al. 2019), and circularization verified by circle_check.py (Meng et al. 2019). All sequences were deposited in GenBank (Table 2).

**Table 3.** Primers used for PCR and sequencing in this study.

**Marker**	**Primer**	**Sequence (5’–3’)**	**Round**	**Source**
COI	LCO1490	GGTCAACAAATCATAAAGATATTGG		Folmer et al. (1994)
	HCO2198	TAAACTTCAGGGTGACCAAAAAATCA		
28S	28sF1	GAGTACGTGAAACCGTCTAG		Pérez-Losada et al. (2009)
	28SR1	CGTTTCGTCCCCAAGGCCTC		
AKAP17	AKAP17-F1	AAYTGGGARGTNATGGARAA	Round1	Liu et al. (2025a)
	AKAP17-R1	TCYTTRAACATNARYTTCAT		
	AKAP17-F2	AARATGATHAARCCNGAYCARTT	Round2	
	AKAP17-R2	GCYTTNACRAANCCCATRTAYTC		
FLAD1	FLAD1-F1	GGNCCNACNCAYGAYGAYAT	Round1	
	FLAD1-R1	TTNGGRTGNGTRTTYTCCAT		
	FLAD1-F2	TGYAARGCNTTYTTYGGNACNGA	Round2	
	FLAD1-R2	TTNACNCKCATRAAYTCNGGCCA		

### Phylogenetic inference

Phylogenetic analyses were performed on a concatenated dataset of 13 protein-coding genes (PCGs) extracted from mitogenomes by a script of gbseqextractor_v2.py (Meng et al. 2019). *Mesenchytraeus anisodiverticulus* Chen, 2012 and *Enchyraeus irregularis* Vejdovsky, 1879 were used as outgroup. Sequences were aligned using MAFFT (Rozewicki et al. 2019), and the best-fit nucleotide substitution model was selected with jModelTest-NG (Darriba et al. 2020) under the Akaike Information Criterion (AIC), and the best model is GTR + I + G. Maximum-likelihood (ML) and Bayesian inference (BI) trees were reconstructed using RAxML-NG (Kozlov et al. 2019) and MrBayes 3.2.6 (Ronquist et al. 2012), respectively. ML nodal support was assessed by 1,000 rapid bootstrap replicates, with bootstrap values (BV) ≥ 70 considered well supported. BI comprised two million MCMC generations, with convergence confirmed by split frequencies < 0.01 and check ESS values > 200 in Tracer v. 1.7.5 (Rambaut et al. 2018). Posterior probabilities (PP) ≥ 0.95 were accepted as strong support. The resulting trees were visualized in FigTree v. 1.4.4 (https://tree.bio.ed.ac.uk/software/figtree/).

### Species delimitation

Species boundaries were assessed using Assemble Species by Automatic Partitioning (ASAP, Puillandre et al. 2021), the Generalized Mixed Yule Coalescent (GMYC; Drummond et al. 2012), and Bayesian Phylogenetics and Phylogeography (BPP; Yang and Rannala 2010). Single-locus analyses of COI and 28S were performed with ASAP (K2P distances, Pmin = 0.001, *P*_max_ = 0.1, steps = 10, *X* = 1.0, 20 bins) and GMYC (ultrametric trees inferred in BEAST v. 1.7.5 under Yule prior and strict clock; GMYC performed using the splits package in R; Ezard et al. 2017). Multi-locus validation used BPP v. 2.2 (Yang and Rannala 2010) on the four-gene dataset under the multispecies coalescent model, with an A00 analysis (estimating θ and τ) and an A10 analysis (species delimitation). Prior settings: θ ~ G (3, 1000); root τ0 ~ G (2, 2000); other τ parameters assigned a uniform Dirichlet distribution (Yang 2002, 2015; Rannala and Yang 2003; Flouri et al. 2018).

## Results

### Taxonomy

**Family Moniligastridae Claus, 1880**

#### Genus *Drawida* Michaelsen, 1900

##### Drawida gisti gisti

Taxon classificationAnimaliaMoniligastridaMoniligastridae

Michaelsen, 1931

A4EE0A76-F11C-5CFE-BDAC-28A992F717F9

Figs 1, 3

Drawida gisti : Michaelsen 1931: 525.
Drawida gisti f. *typica*: Chen 1933: 195–200.
Drawida gisti : Gates 1935: 2–3.
Drawida gisti : Gates 1939: 406–408.
Drawida gisti gisti : Blakemore et al. 2010: 1; Blakemore and Kupriyanova 2010: 8.

###### Material examined.

• Five clitellates (SDRZ_03, SDRZ_04, L668_09_01, DLS_35, SDHZ4_01). • SDRZ_03, SDRZ_04: Rizhao, Shandong, China, Oct-2025, collected by Jiangni Li; • L668_09_01: Xinglong, Hebei, China, Jul-2025, collected by Huifeng Zhao; • DLS_35: Dongling Mountain Beijing, China, Jul-2025; • SDHZ4_01: Heze, Shandong, China, Oct-2025, collected by Yanmeng Bi. All specimens were preserved in the Hebei Key Laboratory of Animal Diversity, Langfang Normal University, Hebei, China (C-HLU).

###### External characteristics (Fig. 1).

Length 48–66 mm, diameter 5.0–7.0 mm. Segments 135–194. The setae begin on II and are closely paired; *aa* slightly less than *bc*. Setae clearly visible. Prostomium prolobous. Dorsal pores absent. The clitellar coloration variable, extending across segments IX/X–XIII/XIV. A pair of spermathecal pores are broad transverse slits on 7/8, at the middle of the *cd*, with the anterior and posterior margins swollen so that the pores appear elevated. The female pores are very minute, narrow transverse slits on 11/12, the slits in *ab*, external invisible. On 10/11 there is a larger transverse slit, in which a pair of penes is situated in *bc*, slightly nearer to *b* than to *c*. The true male pore is much smaller and opens at the ventral end of the penis as a longitudinal slit, about half the length of the penis. The penis of the examined specimens is 2.827 mm in length.

**Figure 1.**
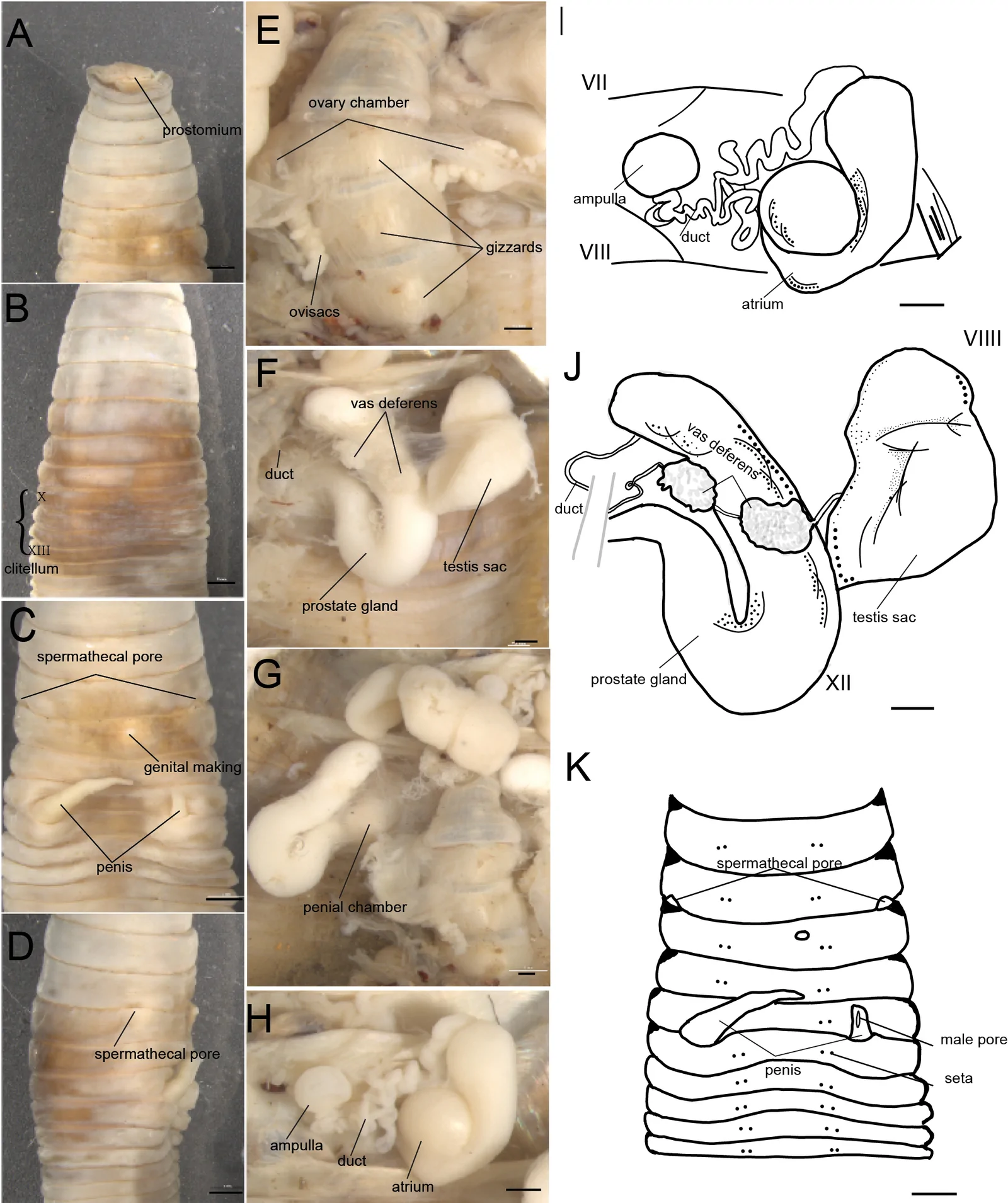
*Drawida gisti gisti* (SDRZ_03). **A**. Ventral view of the prostomium; **B**. Dorsal view of clitellum region; **C, K**. Ventral view of male pores, spermathecal pores, seta, and genital markings; **D**. Lateral view of the spermathecal pores, penis, and clitellum; **E**. Dorsal view of gizzards, ovisacs, and ovary chamber; **F, J**. Dorsal view of right testis sac, with vas deferens and prostate gland; **G**. Dorsal view of left testis sac and penial chamber; **H, I**. Dorsal view of right spermathecae. Scale bars: 1.0 mm (**A, B**); 0.5 mm (**C–K**).

###### Internal characteristics (Fig. 1).

Hearts from V to IX. Septa 5/6, 6/7, 7/8 and 8/9 thickened. Three gizzards in IX/X–XV/XVII/XVIII, occupying seven or nine segments. Testis sacs in two pairs, kidney-shaped, the concave side directed ventrally; in IX–X, or IX, or IX–XI, unconstricted by 9/10, but enveloped by fine membranes and fixed in the ventral body cavity. From the middle of each testis sac near the ventral median line a slender vas deferens emerges, first coiled into a tight bunch of loops on the anterior face of 9/10, then into another tight bunch on the posterior face; the anterior bunch smaller. The vas deferens finally opens on the ventral body wall of segment X. Prostate glands are in two parts: glandular portion and the prostatic duct. The glandular portion is covered with an outer layer of glandular tissue; when the outer layer is removed, a firm, slender, glossy central body is visible (Fig. 3), gradually narrowing toward the distal end; near the penial chamber the prostatic duct is visible, narrower than the glandular portion and lacking the outer granular tissue, 1.048 mm in length, finally opening at the dorsal center of the penial chamber. The penial chambers are in 10/11, projecting conspicuously into the body cavity, however, this projection is not present in all individuals (in specimen SDRZ_03 the right penial chamber was not observed). The prostates are 7.638 mm in length (including the glandular portion and the terminal prostatic duct). The middle portion is bent into a sort of C-shape and is placed around the penial chambers. The ovary chambers present, ovaries in X, ovisacs in X–XIII/XVI/XVIII, The spermathecae are in VIII; the ampulla small, 0.849 × 0.716 mm (SDRZ_03); the duct tightly coiled, of moderate length, gradually widening toward the ampulla; the atrium large, comma-shaped, 2.373 × 1.726 mm (SDRZ_03). On the inner side of the atrium there is a characteristic swelling, this swelling is an intrinsic part of the atrium (a localized dilation of its wall), and should not be confused with the accessory gland, although it superficially resembles the latter but is noticeably larger.

###### Distribution.

The eastern China (except northeastern China).

###### Remarks.

*Drawida gisti gisti*, *D. anqingiana*, and *D. gisti nanchangiana* share several morphological similarities: all three species possess three gizzards, and their prostates terminate in a slender duct connecting to the penial chamber, and ovarian chambers surrounded by septa, and spermathecal ampulla are relatively small with coiled ducts. The differences among the three are as follows: the prostate of *D. gisti nanchangiana* measures 2.0–2.5 mm in length, and *D. gisti gisti* is 7.6 mm, and reaching a maximum of 2.0 mm of *D. anchingiana*; the spermathecal atrium of *D. gisti gisti* is a larger (2.4 mm in length), that of *D. anchingiana* is a smaller, short-cylindrical to globose structure reaching a maximum of 2.0 mm, and that of *D. g. nanchangiana* is thumb-shaped and small. Prostate morphology differs substantially among *D. gisti gisti* and the other species of the complex. In *D. gisti gisti*, *D. cheni*, *D. linhaiensis*, *D. sinica*, and *D. sulcata*, the prostates are cylindrical, relatively long, but curved in various configurations. In *D. syringa*, however, the prostates are shorter and possess an additional outer glandular layer; comparative measurements are given in Table 1.

Moreover, there is variation in penis length within *D. gisti gisti*. SDRZ_03 and SDRZ_04 possess long, well-developed penises, which is consistent with *D. gisti*.The penes of the remaining specimens are relatively small and lamellate, presumably due to morphological variation induced by differences in habitat environment. Additionally, these specimens exhibit some color variation, yet their internal morphology is highly uniform.

##### Drawida xuqini

Taxon classificationAnimaliaMoniligastridaMoniligastridae

Qiao & Zhao sp. nov.

EA8B5612-20BA-5C52-8E01-E9CB3F96DE1E

https://zoobank.org/BE7F4BB7-F944-44BF-AA5D-F70CD7E358EA

Fig. 2

###### Material examined.

***Holotype***: • One clitellate (mature) specimen (345R_39) (87 mm in total length, dissected), Weiyuan Park, Shanhaiguan District, Qinhuangdao Prefecture, Hebei, China, 40.0139°N, 119.7485°E, 21-Aug-2019, collected by Yufeng Zhang. ***Paratypes***: • Five clitellates (345R_33, 345R_34, 345R_37, 345R_38, 345R_40), data the same as the holotype. All specimens were preserved in C-HLU.

###### Diagnosis.

Length 48–87 mm, diameter 4.5–7.0 mm. Segments 160–181. Body light gray in 95% ethanol. Prostomium prolobous, dorsal pores absent. Clitellum pale pink, on segments X–XIII. Gizzards 3–5 in number. Vas deferens very short and uncoiled, a key character distinguishing it from most congeners in the DiSC. Prostates distinctly M-shaped, having a very slender terminal prostatic duct. Penes thick, with swollen base and blunt rounded apex; male pores minute, opens on the ventral face of the penis. Ovaries enclosed in ovarian chambers in segments XI–XII. Spermathecae situated in segment VIII, with large ampulla and small elliptical atrium.

**Figure 2.**
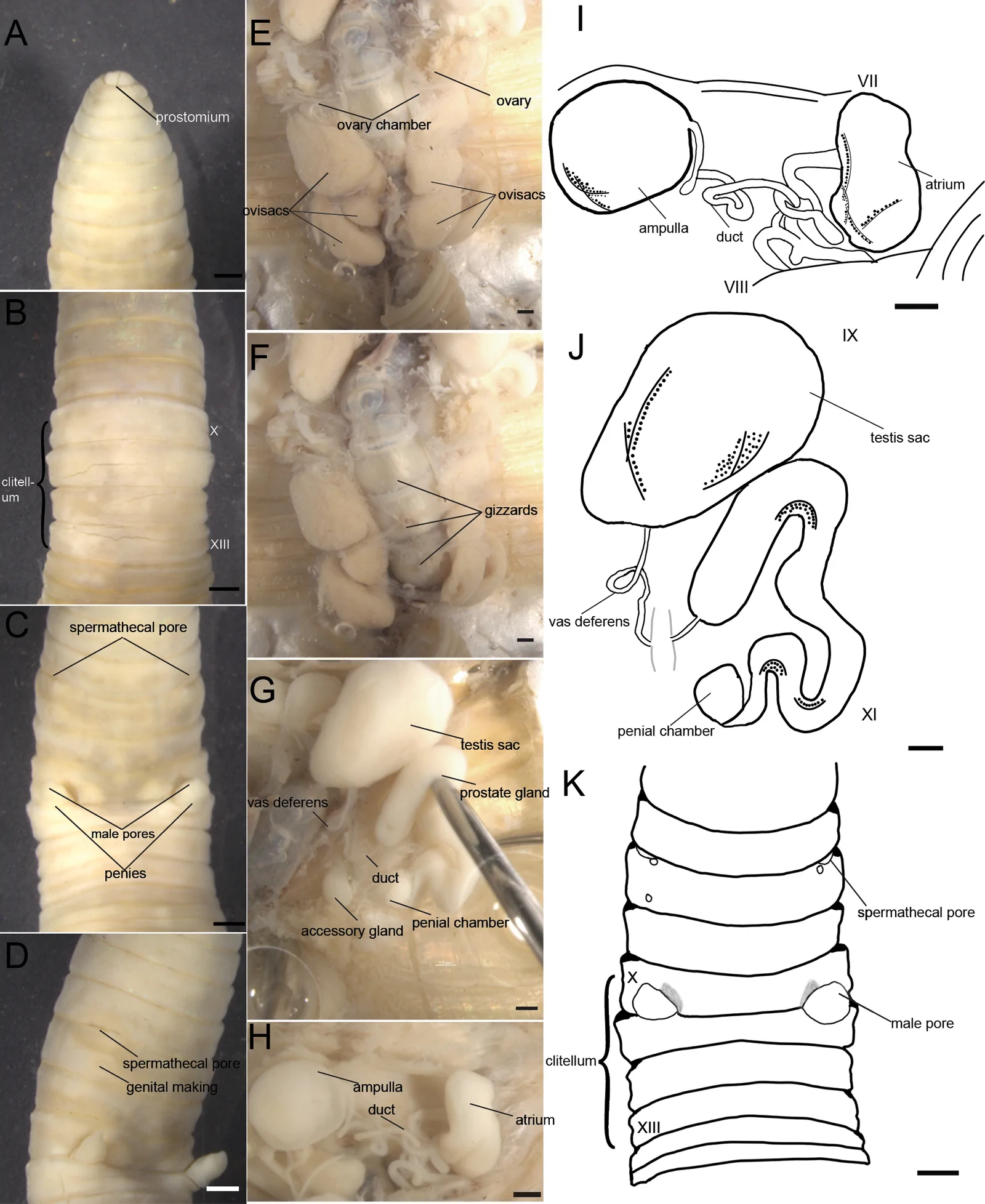
*Drawida xuqini* sp. nov., holotype (345R_39). **A**. Ventral view of the prostomium; **B**. Dorsal view of clitellum region; **C, K**. Ventral view of male pores, spermathecal pores and genital markings; **D**. Lateral view of the spermathecal pores, penis and clitellum; **E**. Dorsal view of ovisacs, ovaries and ovary chamber; **F**. Dorsal view of gizzards; **G, J**. Dorsal view of right testis sac, with vas deferens and prostate gland; **H, I**. Dorsal view of right spermathecae. Scale bars: 1.0 mm (**A–D, K**); 0.5 mm (**E–J**).

**Figure 3.**
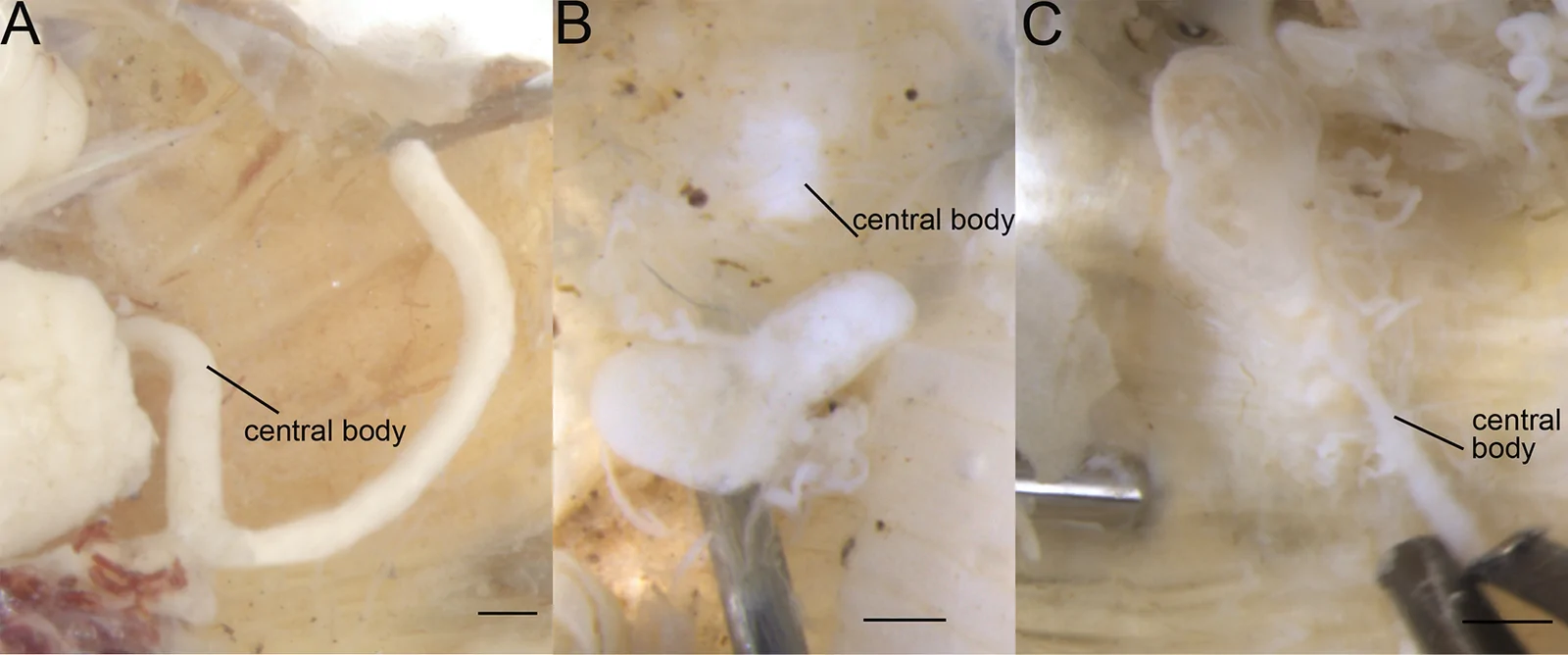
Morphology of the prostatic central body. **A**. *Drawida gisti gisti* (SDRZ_04); **B**. *Drawida wuxiensis*; **C**. *Drawida xinjiangensis*. Scale bars: 0.5 mm.

###### External characteristics.

Holotype: length 87 mm, diameter 7.0 mm. Segments 164. The setae begin on II and are closely paired; *ab*:*cd*:*aa*:*bc* = 1:1*ab*:10*ab*:10*ab*. Prostomium prolobous. Dorsal pores absent or invisible. Body color brown, head greyish blue, clitellum pale pink. Clitellum X–XIII. The spermathecal pores are narrow transverse slits on 7/8, at *bc*, close to *c* than *b*. The female pores are very minute, narrow transverse slits on 11/12, external invisible. On 10/11 there is a larger transverse slit, in which a pair of penes are situated in *bc*, slightly nearer to *b* than to *c*. The true male pore is much smaller and opens on the ventral face of the penis but is inconspicuous, no longitudinal or transverse slit observed. The left penis is 0.929 mm in length; the right is 1.066 mm in length. The penes of this new species are rather thick, swollen at the base and gradually tapering and the apex blunt resembling a triangular cone. Genital markings present, irregular and three in number, on segment VIII. The genital markings are mainly concentrated around the spermathecal pores.

###### Internal characteristics.

Holotype: hearts from V to IX. Septa 5/6, 6/7, 7/8 and 8/9 thickened. The gizzards three to five, in IX–XVI, XII/XIII–XVIII or XI–XVII, occupying six, seven or eight segments. The testis sacs two pairs, 2.362 × 3.545 mm in size, elliptical; in IX–X, or IX/X, or IX–XI, occupying one to two segments, occasionally three, unconstricted by 9/10. A very short, uncoiled vas deferens arises from the margin at two-thirds of the testis sac, finally opening on the ventral body wall of segment X, 2.977 mm in length. One pair of prostates are M-shaped, 11.614 mm in length, only the glandular portion enveloped by granulations (external glandular layer), and when this outer layer is removed a smooth central body is visible. The prostate width gradually narrows, 0.828 mm at the apex and 0.302 mm at the distal end. On the inner side of the prostate near the ventral median line there are one or two accessory glands. The penial chambers in 10/11. A very short prostatic duct arises from the apex of the prostate, 0.437 mm in length, lying close to the opening of the vas deferens. The ovaries are on each side of the gizzard, in XI–XII, inverted U-shaped; the septa form ovarian chambers, and when these are removed the white ovaries are visible. The ovisacs appear as brown sacs covering the gizzard, extending to segment XXI. The spermathecae are in VIII; the ampulla large, milky white, 1.497 × 1.694 mm in size; the atrium small, elliptical, pale yellow, 1.853 × 0.859 mm in size, situated on the septum 7/8. The duct tightly coiled, gradually widening from the end of the ampulla and finally ending in the middle of the atrium; the anterior portion 0.129 mm in width, the posterior portion 0.173 mm in width, the duct about 10.0 mm in length.

###### Variation.

In the paratypes, the genital markings are irregular, unpaired, and scattered on segments VII–XI. The number and position of gizzards vary as follows: specimen 345R_34 has three gizzards in segments 13–18; 345R_33 has five gizzards in segments 9–16; 345R_38 has four gizzards in segments 9–16; 345R_37 has three gizzards in segments 12–18; and 345R_40 has three gizzards in segments 11–17. Thus, the gizzard number ranges from three to five, and the segmental distribution ranges from nine to 18 among the paratypes. This variation is considered intraspecific, as is commonly observed in the genus *Drawida*. The testis sacs are two pairs, elliptical, measuring 2.362 × 3.545 mm, and occur in IX–X, or at IX/X, or in IX–XI, occupying one or two segments (occasionally three), and are not constricted by septum 9/10. The vas deferens ranges from 2.208 to 3.113 mm in length. The prostates are one pair, M-shaped, 4.705–11.614 mm long. The spermathecal ampulla is large, milky white, about 1.43 × 1.47 mm; the atrium is small, elliptical, pale yellow, about 1.585 × 0.859 mm. The ovisacs appear as brown sacs covering the gizzard and extend to segments XVI–XX.

###### Etymology.

The specific epithet *xuqini* is a patronymic genitive noun formed, in honor of Mr Qin Xu, the first author of “Terrestrial Earthworms (Opisthopora: Oligochaeta) of China”.

###### Distribution.

Known only from the type locality (Qinhuangdao, Hebei).

###### Remarks.

*Drawida xuqini* sp. nov. is readily distinguished from other members of the *D. gisti* species complex by a combination of clear morphological characters. The penis is thick, with a swollen base and a blunt, rounded apex, whereas in *D. gisti gisti* it is slender, elongated, and tapers to a sharp tip. The new species possesses 3–5 gizzards located in segments 9–18 (with intraspecific variation), while *D. gisti gisti* and most other species consistently have three gizzards. The vas deferens is very short and entirely uncoiled, a unique character among the complex (vs long and coiled in *D. gisti gisti*, *D. anchingiana*, *D. g. nanchangiana*, *D. linhaiensis*, *D. sinica*, and *D. syringa*; short but coiled in *D. sulcata* and *D. cheni*). The spermathecal atrium is elliptical and large in the new species (comma-shaped in *D. gisti gisti*), and the prostate is M-shaped without a slender terminal duct. The body color in alcohol is light gray (vs reddish brown in *D. gisti gisti*).

### Phylogenetic analyses

**Figure 4.**
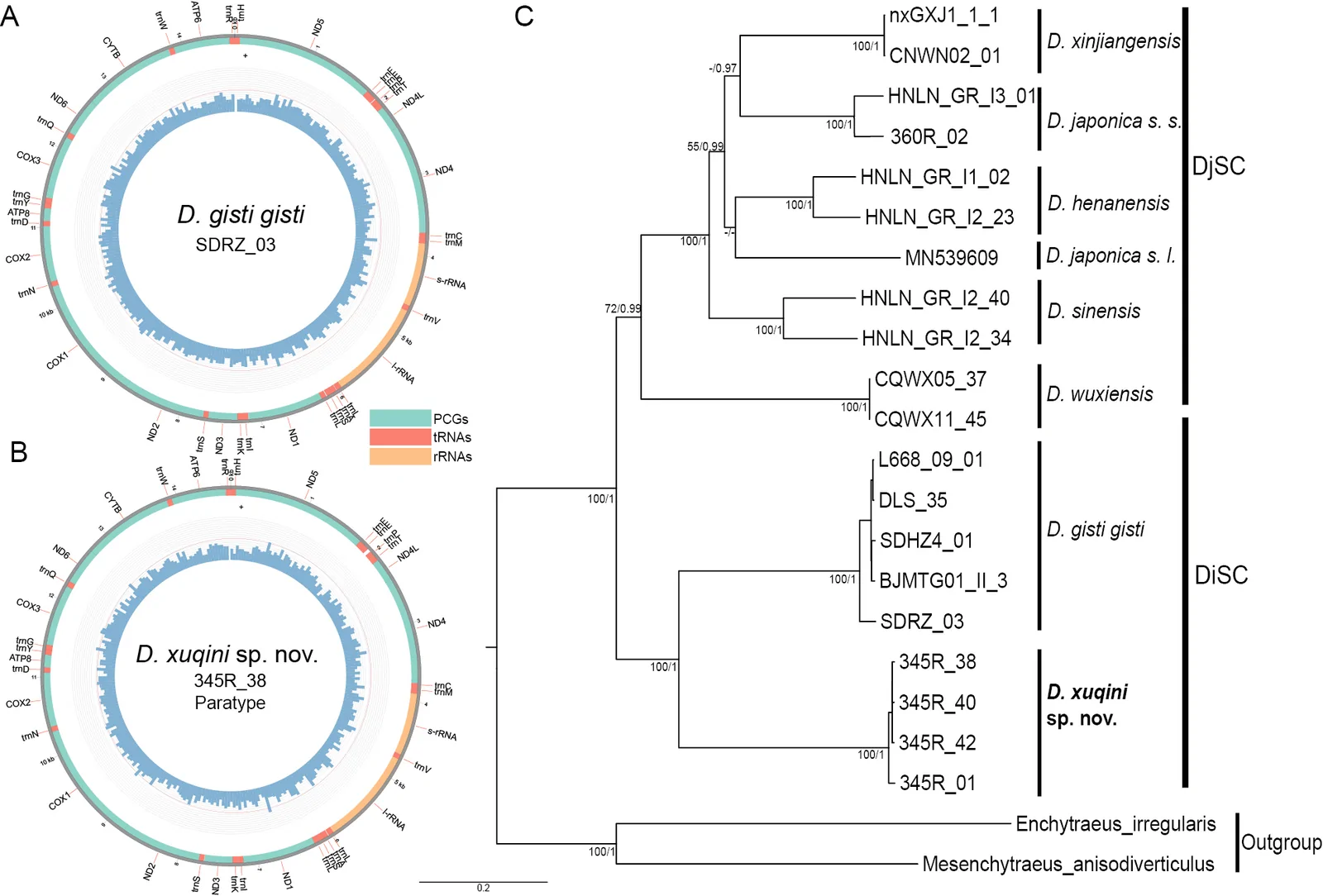
**A, B**. Mitogenomes of *Drawida gisti gisti* and *D. xuqini* sp. nov.; **C**. Phylogenetic relationship of *D. xuqini* sp. nov. and other species in DjSC and DiSC using 13 coding genes of mitogenomes. Numbers near branches indicate the maximum likelihood bootstrap support/Bayesian posterior probabilities. Values with a bootstrap value below 50 and a posterior probability lower than 80 have been omitted. Orange tips showed the new species.

The mitogenomes of *D. gisti gisti* and *D. xuqini* sp. nov. are 14,647–14,648 bp and 14,668–14,750 bp in length, respectively, and all of them contain the typical mitochondrial 37 genes: 13 protein-coding genes (PCGs), two ribosomal RNA genes (rRNAs), and 22 transfer RNA genes (tRNAs). (Fig. 4A, B). The control region (CR) is absent in both species examined here, unlike its typical presence in earthworm mitogenomes (Zhao et al. 2022) but similar to some species of the *Drawida japonica* species complex (DjSC) (Zhang et al. 2016; Qiao et al. 2026a).

Phylogenetic analysis based on the concatenated 13 PCGs (Fig. 4C) shows that DiSC and DjSC forms two independent well-supported monophyletic groups. However, the lineage of MN539609 identified as *D. gisti* (Liu et al. 2020) is clustered in DjSC. Within this, the DiSC, four tips of *D. xuqini* sp. nov. are clustered as a distinct lineage with robust support in both ML and BI analyses (ML Bootstrap value = 100, BI Posterior probability = 1.00). The same situation of five individuals of *D. gisti gisti*. However, SDRZ_03 shows genetic differences with the other four individuals.

### Molecular species delimitation

**Figure 5.**
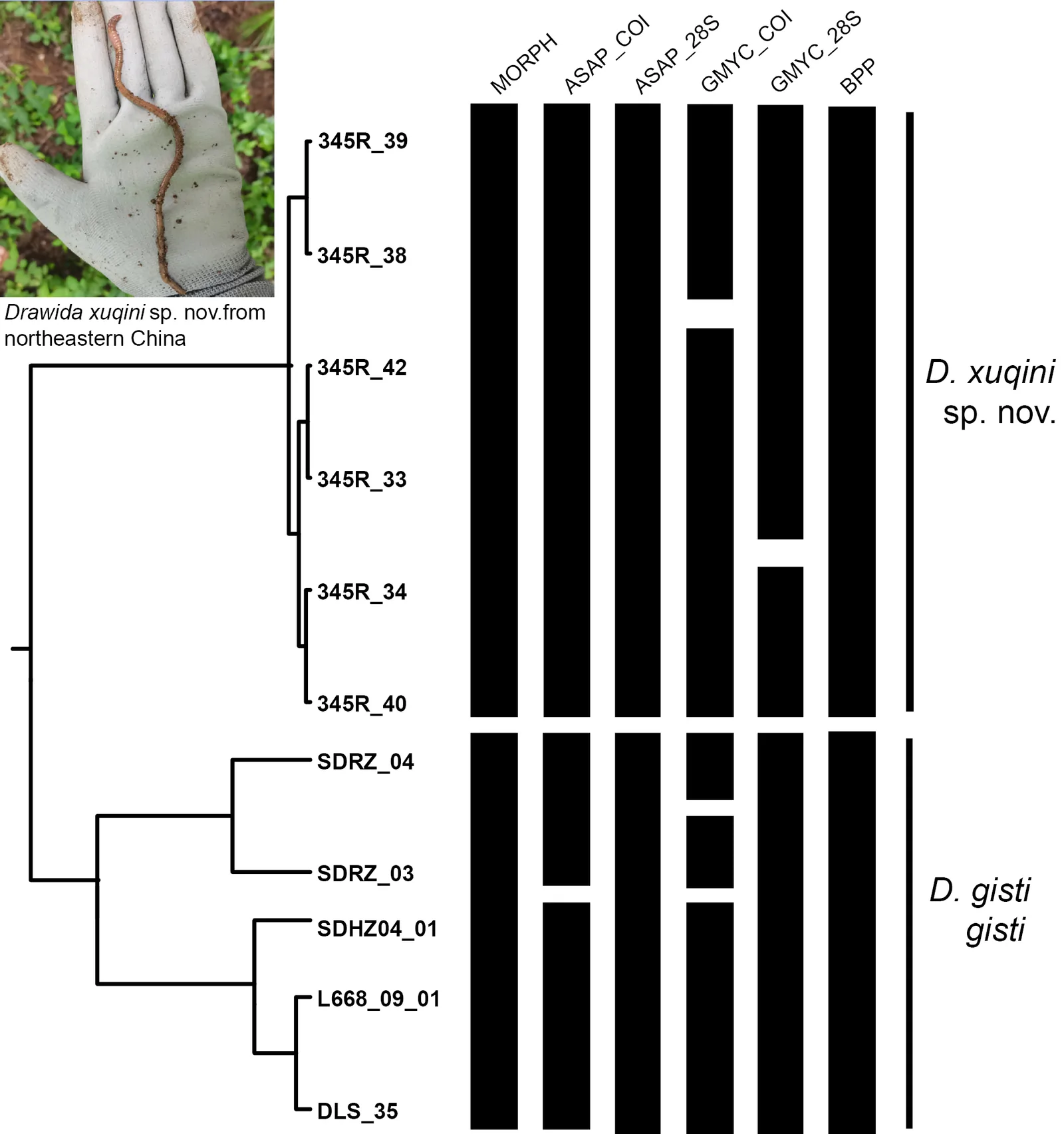
Species delimitation of the *Drawida gisti* species complex. The black vertical bar shows the number of species delimited by morphological characteristics and molecular data. The upper left shows field photographs of the new species.

Three methods—ASAP, GMYC, and BPP—were employed to delimit molecular operational taxonomic units (MOTUs) (Fig. 5). ASAP (ASAP score 1.50) based on COI split *D. gisti gisti* into two MOTUs, whereas GMYC based on the same marker further subdivided *D. gisti gisti* into three MOTUs and split *D. xuqini* sp. nov. into two MOTUs. For the 28S dataset, ASAP (ASAP score 1.00) results are the same as morphological evidence (support *D. gisti gisti* and new species are independent), but GMYC recovered two MOTUs within *D. xuqini* sp. nov.

BPP analysis of the four-locus dataset consistently distinguished *D. gisti gisti* and *D. xuqini* sp. nov. as two distinct species congruent with morphological evidence. The K2P distances based on COI further supported the genetic integrity of the new species: intraspecific divergence ranged from 0 to 0.5%, whereas distances between *D. xuqini* sp. nov. and *D. gisti gisti* fell between 19.4% and 22.0% (Table 4), well above the 15% interspecific threshold routinely applied in earthworm taxonomy (Dong et al. 2019; Chang et al. 2024; Zhao et al. 2025). The subdivision of *D. gisti gisti* by single-locus methods suggests potential cryptic diversity within this nominal species; however, expanded molecular sampling across the entire DiSC is required to test this hypothesis and clarify species boundaries. For detailed results of molecular species delimitation, see the Suppl. materials 1–3.

**Table 4.** The COI genetic distance of K2P of groups of *Drawida* (values are percentages; intraspecific genetic distances are given in parentheses).

	**Species**	**1**	**2**	**3**	**4**	**5**
1	*D. xuqini* sp. nov.	(0–0.5)				
2	*D. gisti gisti*	19.4–22.0	(0.3–5.7)			
3	*D. henanensis*	19.9–23.5	22.6–27.1	(0.–5.7)		
4	*D. japonica* s. s.	23.3–24.3	22.3–23.4	19.2–22.8	(0–0.2)	
5	*D. sinensis*	22.3–23.8	20.8–22.0	20.2–22.5	17.6–22.2	(13.3)

## Discussion

Morphological examinations reveal considerable external plasticity in *Drawida gisti gisti*, with body color and size varying markedly among specimens from different localities. This external plasticity contrasts with the relatively conserved internal characters, which show only minor variations across specimens. Such disparity complicates species delimitation in *Drawida*, underscoring the necessity of integrative taxonomic approaches (Dayrat 2005; Padial et al. 2010; Liu et al. 2025a, 2025b). For example, mitogenome MN539609 was previously misidentified as *D. gisti* by Liu et al. (2020), likely due to insufficient morphological expertise and the lack of authentic DNA sequences for this species.

Notably, specimens with divergent penial morphology from Rizhao populations showed K2P genetic distances of 4.8–5.7% from typical *D. gisti gisti* populations, and species-delimitation analyses using ASAP and GMYC (based on COI) all recovered them as distinct evolutionary lineages. However, it should be noted that GMYC tends to separate biological species into several OTUs due to excessive lineage splitting (Goulpeau et al. 2022), and its application to mitochondrial COI data often yields results incongruent with other delimitation methods (Liu et al. 2025b). Among the northern specimens examined, only SDRZ_03 and SDRZ_04 exhibited penial morphology consistent with the historical description of *D. gisti* (Michaelsen 1931); the remaining specimens matched *D. gisti* in most other internal and external features except for the penis. This suggests that the northern populations likely do not represent simple morphological variants of *D. gisti* from northward dispersal but rather may constitute cryptic species or novel taxa within the complex awaiting further validation through expanded sampling.

The discovery of *D. xuqini* sp. nov. at Shanhaiguan in northeastern China hints at an expansion of distribution in DiSC. The Shanhaiguan population may represent a peripheral isolate that diverged through geographic isolation and local adaptation under different climatic regimes. Conversely, given the wide distribution of allied taxa across eastern China, additional cryptic species may await discovery in the southern portion of the range. Both hypotheses remain to be tested through denser sampling across the northern and southern provinces of China.

## Supplementary Material

XML Treatment for Drawida gisti gisti

XML Treatment for Drawida xuqini

## Additional information

### Artificial Intelligence (AI) use

The authors accept full responsibility for the content of the manuscript, including the disclosure of any use of AI.

No AI tools were used in the preparation of this manuscript.

### Author ORCIDs

S. Qiao https://orcid.org/0009-0002-9964-1352

J. Li https://orcid.org/0000-0003-1642-0328

Y. Zhang https://orcid.org/0000-0002-4066-6355

N.M. Aspe https://orcid.org/0000-0003-0025-6017

H. Zhao https://orcid.org/0000-0003-4243-9671

### Data availability

All of the data that support the findings of this study are available in the main text or Supplementary Information.

## Supplementary materials

Supplementary material 1 GMYC species delimition results Data type pdf Explanation note **fig. S1**. GMYC species delimition results based on COI (**A**) and 28S (**B**). https://binary.pensoft.net/file/1795862This dataset is made available under the Open Database License (http://opendatacommons.org/licenses/odbl/1.0/). The Open Database License (ODbL) is a license agreement intended to allow users to freely share, modify, and use this Dataset while maintaining this same freedom for others, provided that the original source and author(s) are credited. Saiyu Qiao, Jiangni Li, Yufeng Zhang, Nonillon M. Aspe, Huifeng Zhao

Supplementary material 2 The result of molecular species delimitaiton Data type xlsx Explanation note **table SS1**. The result of molecular species delimitaiton of ASAP on COI and 28S in *Drawida gisti gisti* and *D. xuqini* sp. nov. https://binary.pensoft.net/file/1795863This dataset is made available under the Open Database License (http://opendatacommons.org/licenses/odbl/1.0/). The Open Database License (ODbL) is a license agreement intended to allow users to freely share, modify, and use this Dataset while maintaining this same freedom for others, provided that the original source and author(s) are credited. Saiyu Qiao, Jiangni Li, Yufeng Zhang, Nonillon M. Aspe, Huifeng Zhao

Supplementary material 3 BPP related files Data type rar https://binary.pensoft.net/file/1795864This dataset is made available under the Open Database License (http://opendatacommons.org/licenses/odbl/1.0/). The Open Database License (ODbL) is a license agreement intended to allow users to freely share, modify, and use this Dataset while maintaining this same freedom for others, provided that the original source and author(s) are credited. Saiyu Qiao, Jiangni Li, Yufeng Zhang, Nonillon M. Aspe, Huifeng Zhao
