# Supplementary material for: Establishment of the Drawida gisti species complex and description of a new species from northeastern China (Oligochaeta, Moniligastridae)
Source: Zookeys. 2026 Oct 1;1293:327–46. doi: 10.3897/zookeys.1293.204408 (PMC13650586; doi:10.3897/zookeys.1293.204408)
Supplement: Supplementary file 1 [file zookeys-1293-327_article-204408__-s001.pdf]

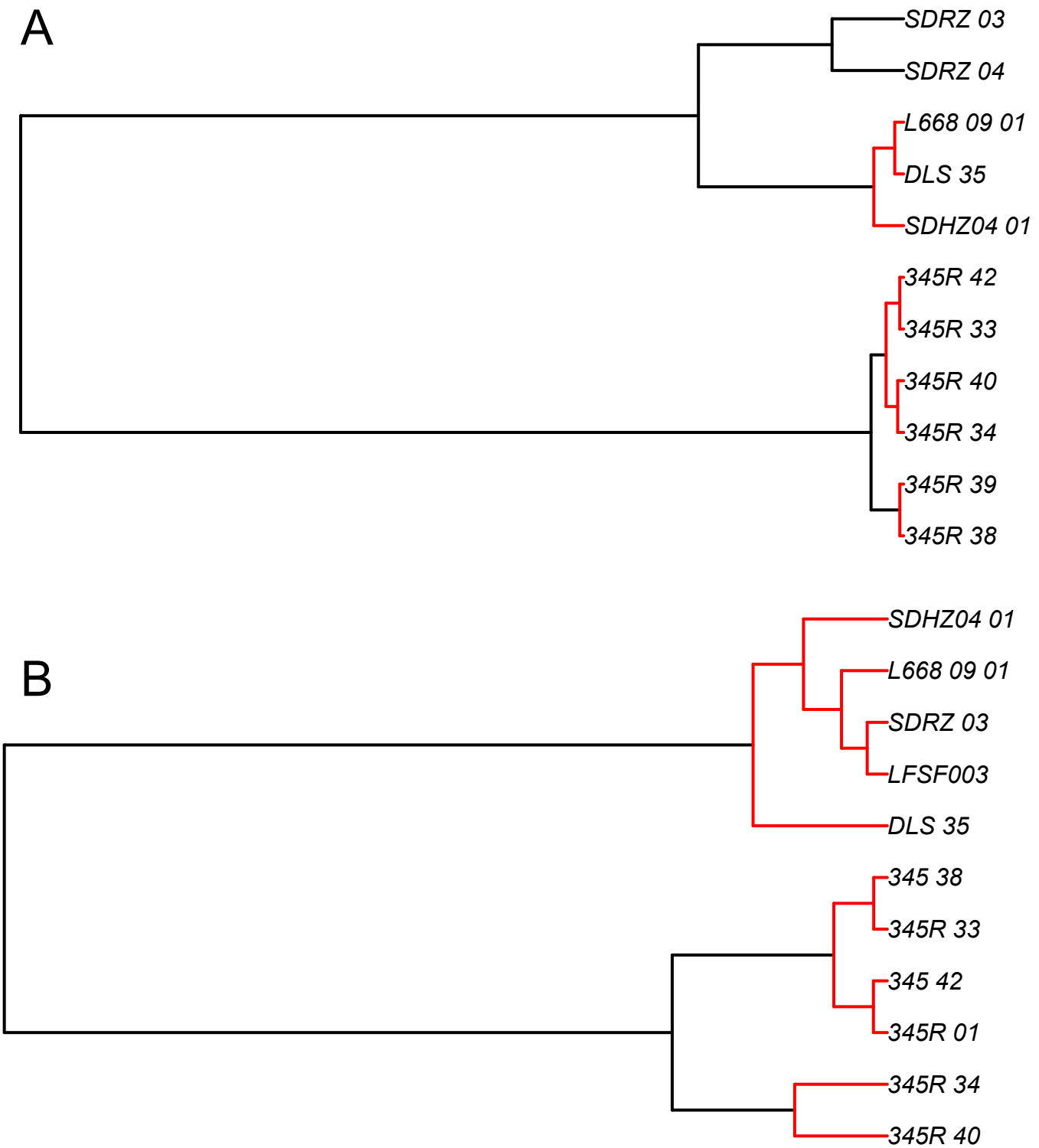

Figure S1. GMYC species delimition results based on COI (A) and 28S (B).
